# Decipering the Molecular Mechanism of ACE2 Regulating A549 Cells

**DOI:** 10.3389/fgene.2021.653725

**Published:** 2021-07-19

**Authors:** Kun Xiao, Licheng Song, Ying Bai, Pengfei Liu, Yuhong Liu, Fei Xie, Lixin Xie

**Affiliations:** Center of Pulmonary and Critical Care Medicine, Chinese People’s Liberation Army (PLA) General Hospital, Beijing, China

**Keywords:** angiotensin-converting enzyme 2, overexpression, regulative mechanism, differentially expressed proteins, functional analysis

## Abstract

Angiotensin-converting enzyme 2 (ACE2) is an aminopeptidase that functions as a part of the renin-angiotensin system (RAS). The RAS pathway plays a crucial role in regulating the local blood flow within a tissue. As a consequence, the role of ACE2 in regulating vasculature properties has been widely appreciated. Additionally, ACE2 has also been reported to show anti-tumorigenic activity. However, the mechanistic basis of this function has remained largely unexplored. In the current study, using a lentivirus-based expression system in lung cancer cells (A549), we show that ACE2 overexpression reduces the viability and migratory potential of cancer cells, highlighting the robust anti-tumorigenic effects of ACE2 function. Moreover, a quantitative proteome-level comparison between ACE2 overexpressed (OE) and empty vector-controlled (NC) cells reveals a large number (227) of differentially expressed proteins (DEPs) that may have contributed to this phenomenon. Functional enrichment of these DEPs has uncovered that most of them perform binding activities and enzymatic reactions associated with metabolic pathways and various post-transcriptional gene expression regulatory mechanisms. Besides, cellular component analysis reveals that the DEPs function across a range of compartments within a cell with a relatively heterogeneous distribution. Our study, therefore, supports the previously established anti-tumorigenic effects of ACE2 overexpression in lung cancer cells. An analysis based on comprehensive, unbiased, and quantitative proteomics, we have provided a rigorous mechanistic explanation for its functions.

## Introduction

The renin-angiotensin signaling (RAS) pathway is a critical homeostatic modulator of vascular functions, including the regulation of blood pressure, blood volume, and natriuresis (Paz Ocaranza et al., 2020). The RAS is composed of multiple proteases and effector peptides that are crucial to dynamically modulate the local properties of a vascular function within a tissue. For example, the end product of the RAS pathway is angiotensin II (Ang II), a widely studied effector peptide. It is produced from its precursor polypeptide angiotensin I (Ang I) by the action of a dipeptidase named angiotensin-converting enzyme (ACE) (Tikellis et al., 2012). However, to ensure a coordinated stimulus-specific response, the RAS contains a set of critical enzymes that function antagonistically to each other (Paz Ocaranza et al., 2020). Angiotensin-converting enzyme 2 (ACE2), a homolog of the previously described ACE, produces vasodilatory peptides Angiotensin1-7, therefore, counterbalancing the vasoconstrictor functions of Ang II (Hamming et al., 2007; Paz Ocaranza et al., 2020). While conventionally, ACE and Ang II have drawn much attention due to their involvement in multiple pathogenic conditions, more recent studies have shifted focus to understand the functions of ACE2 as a potential target for therapeutics in the RAS-related pathologies.

ACE 2 is an 805 amino acid-long type 1 integral membrane glycoprotein that is actively expressed in most tissues (Prabakaran et al., 2004). The highest level of expression, however, is found in the heart, lung, kidney, and endothelium (Dai et al., 2020; Ng et al., 2020). While Ang II is its major substrate peptide, ACE2 can cleave amino acids from the C-terminus of multiple other peptides such Ang I, vasoactive bradykinin, des-Arg Kallidin, Apelin 13, 36, and others (Tikellis et al., 2012). Recently, ACE2 has drawn further attention due to its ability to bind to SARS-CoVs. Both SARS-CoV 1 and 2 can bind to ACE2 through the receptor-binding domain of the spike proteins on their capsid (Tikellis et al., 2012; Yang et al., 2020). Thus, investigations aimed to alter the vasculature properties in heart, lung, or kidney-related pathologies and to inhibit SARS-CoV entry into the host cell by therapeutically targeting ACE2 hold tremendous potential (Yang et al., 2020).

Additionally, ACE2 and RAS are thought to have crucial roles in tumor growth and development. Significant changes in ACE2 levels are observed in various types of cancers documented in multiple database (Chai et al., 2020; Dai et al., 2020). For example, while lung EGFR-mutant adenocarcinoma cells express elevated levels of ACE2, liver cancer patient samples show a significant drop in the expression of the protein (Yamaguchi et al., 2017; Dai et al., 2020). In fact, in a large majority of the cancers, the ACE2 expression level is found to be reduced (Chai et al., 2020). Also, lower ACE2 expression correlates well with a poorer prognosis of the disease across cancer types. For example, studies in breast cancer cells have revealed that they show elevated metastatic potential due to their lower ACE2 levels compared to non-cancerous control cells (Zhang et al., 2019). Likewise, ACE2 overexpression in human umbilical vascular endothelial cells abrogates their migration and angiogenesis by attenuating the VEGFa and ERK1/2 signaling (Zhang et al., 2019; Ma et al., 2020). In non-small cell lung carcinoma, ACE2 overexpression inhibits TGF-β1-induced epithelial to mesenchymal transition (EMT) and Ang II-driven VEGFa signaling that ultimately lessens cancer cell metastasis (Feng et al., 2010; Qian et al., 2013). Together these studies reveal that ACE2 can antagonize key features of cancer cell functions such as their metastatic and angiogenic potential. These studies also suggest which signaling pathways might be relevant to the anti-tumorigenic actions of ACE2. Yet a comprehensive understanding of the proteome-wide changes due to ACE2 overexpression is lacking, which might prove useful to understand its function further in the context of tumor growth and development.

In the current study, we explored the functions of ACE2 in greater depth in lung cancer cells (A549) using a lentivirus-based overexpression system. We observed a significant reduction in cancer cell proliferation and invasion potential, along with elevated cellular apoptosis, in ACE2 overexpressing lung cancer cell lines. Additionally, a quantitative proteome-level comparison between ACE2 overexpression (OE) and normal control (NC) cells revealed that a large number of proteins (227) were expressed differentially between the two conditions suggesting a profound proteome level changes accompany ACE2 overexpression. Functional enrichment of these DE expressed differentially proteins (DEPs) uncovered most of them harboring binding functions and enzymatic activities associated either with metabolic reactions or gene expression regulatory proteins. Additionally, the cellular component analysis revealed that the DEP functions were associated with cytosolic and extracellular compartments.

## Results

### Establishment of a Lentivirus-Based Overexpression System for ACE2 in Lung Cancer Cells

**FIGURE 1:**
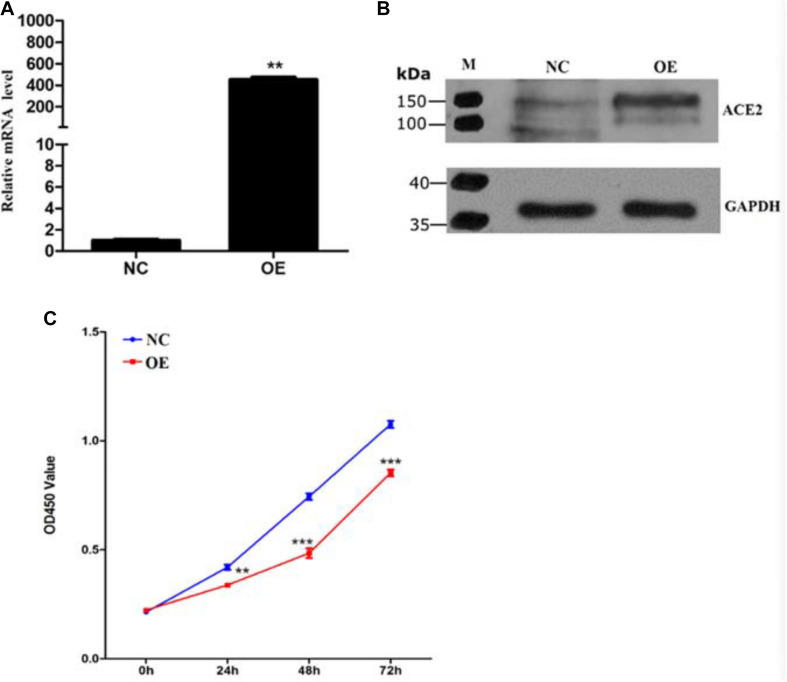
Establishment of ACE2 overexpression cell line and characterization of its growth rate. **(A)** Quantitative expression analysis of *ACE2* mRNA examined by RT-qPCR with *GAPDH* mRNA as an internal reference. Values are normalized to levels observed in cells with expression control vector pcDNA3.1 (*n* = 3 cultures, paired Student’s *t*-test, ± SD). **P < 0.01, ***p < 0.001. **(B)** Immunoblotting for ACE2 from control and ACE2 overexpressing cells with GAPDH as a loading control. **(C)** Optical density measurements at 490 nm indicating the extent of CCK8 uptake from NC (blue) and ACE2 OE (red) A549 cells. Line graphs show the kinetics of cell growth quantified at different time points after plating (*n* = 3 cultures, Student *t*-test, ± SD).

To study the role of ACE2 in tumorigenicity, we decided to overexpress the ACE2 gene stably in the non-small cell lung carcinoma cell line A549 using a lentivirus-based expression system (pcDNA3.1-ACE2). To validate, both qPCR-based quantification of ACE2 mRNA levels and immunoblotting-based quantification of ACE2 protein levels were performed. Real-time qPCR analysis revealed a 500-fold increase in ACE2 mRNA level compared to the empty vector transduced cells, suggesting a successful overexpression of ACE2 in this cell line (Figure 1A). Further, the immunoblotting assay showed a robust elevation in the ACE2 protein level in OE cells compared to the NC ones (Figure 1B). These results indicate a successful establishment of the human mesenchymal stem cell line overexpressing ACE2.

### ACE2-Mediated Regulation of Proliferation and Apoptosis of Lung Cancer Cells

**FIGURE 2:**
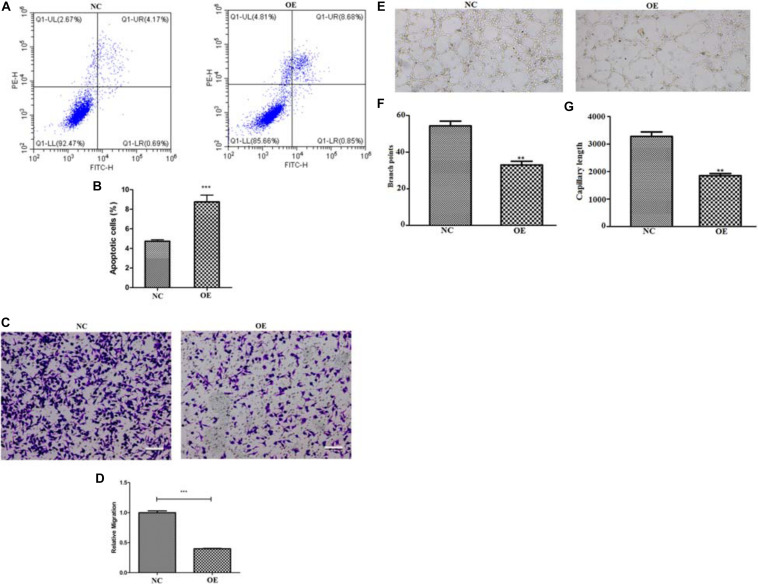
Quantifying apoptosis and migratory potential of ACE2 overexpressing cells. **(A,B)** Flow cytometry analysis, represented by scattered dot plots **(A)** and associated quantification **(B)**, to measure the percentage of apoptotic cells present in either normal control (NC) or ACE2 overexpressing (OE) cells (*n* = 3 cultures, paired Student’s *t*-test, ± SD). **(C,D)** Trans-well migration assay, shown by the representative images of migrated cells **(C)** and associated quantification **(D)**, to measure the migratory potential of either NC or ACE2 OE cells (*n* = 3 cultures, paired Student’s *t*-test, ± SD). **(E)** Tube formation assay shown by representative image and quantification shows the difference in **(F)** branch point and **(G)** capillary length. **P < 0.01, ***P < 0.001.

To assess whether ACE2 overexpression alters the viability of the lung cancer cells, we decided to perform a cell viability assay for both the NC and OE cells in 96 well dishes following various periods of plating (24, 48, and 72 h). A 2 h incubation of cells with CCK8 was performed for both cell types. CCK8 gets converted to a formazan dye because of the dehydrogenase activity present within live cells that can be detected by colorimetric absorbance (Cai et al., 2019). Therefore, the amount of the accumulated dye that can absorb at 490 nm is directly proportional to the number of live cells. Our quantification revealed that the OE cells showed significantly reduced absorbance values at all the time points indicating that the lung cancer cells proliferate at a much slower rate upon ACE2 overexpression (Figure 1C). It is important to note that our quantifications relating cell viability is a steady-state measurement at any given time and can be influenced by both the rate of cell proliferation and the rate of apoptosis. Further, to measure the if the ACE2 overexpression cause programmed cell death we measure the well know markers such as Bax and P53 by western blot. Our results suggest a significant increase in the both Bax and P53 protein levels in the OE cells (Supplementary Figures 1A–C). To decipher whether ACE2 overexpression also increased cell death, we decided to count the number of apoptotic cells in both NC and OE cells. We stained both cultures with Annexin-V conjugated with FITC (apoptosis detection kit, BD Franklin Lakes, NJ) and the number of labeled cells was determined using flow cytometry-based methods. We observed a significant increase in the number of Annexin-FITC labeled cells upon OE compared to NC cells indicating a large elevation in the number of apoptotic cells (Figures 2A,B).

### ACE2 Regulates the Migratory Potential of Lung Cancer Cells

Next, we wondered whether ACE2 overexpression can suppress the migratory potential of the lung cancer cells, as has been shown in the case of breast cancer cells (Zhang et al., 2019). To check, we performed the trans-well migration assay and compared between the two groups of cells. In this assay, two chambers were separated by an 8 μm pore trans-well chamber. About 1 × 10^5^ cells were first placed on the Matrigel-coated upper chamber and the number of the invaded cells that reached the other chamber were fixed and counted. We observed a significant reduction in the number of invading cells upon OE compared to NC cells suggesting that elevated levels of ACE2 could impede the ability of lung cancer cells to migrate (Figures 2C,D). The above pieces of evidence, together, suggest that ACE2 overexpression reduced cellular growth rate and induced robust apoptosis in lung cancer cells while suppressing their migratory potential. We also performed the tube formation assay for the NC and OE cells, we observe a significant reduced branch point and capillary length (Figures 2E–G).

### Deciphering the Molecular Mechanisms of ACE2 Function Using Quantitative Proteomic Analysis

**FIGURE 3:**
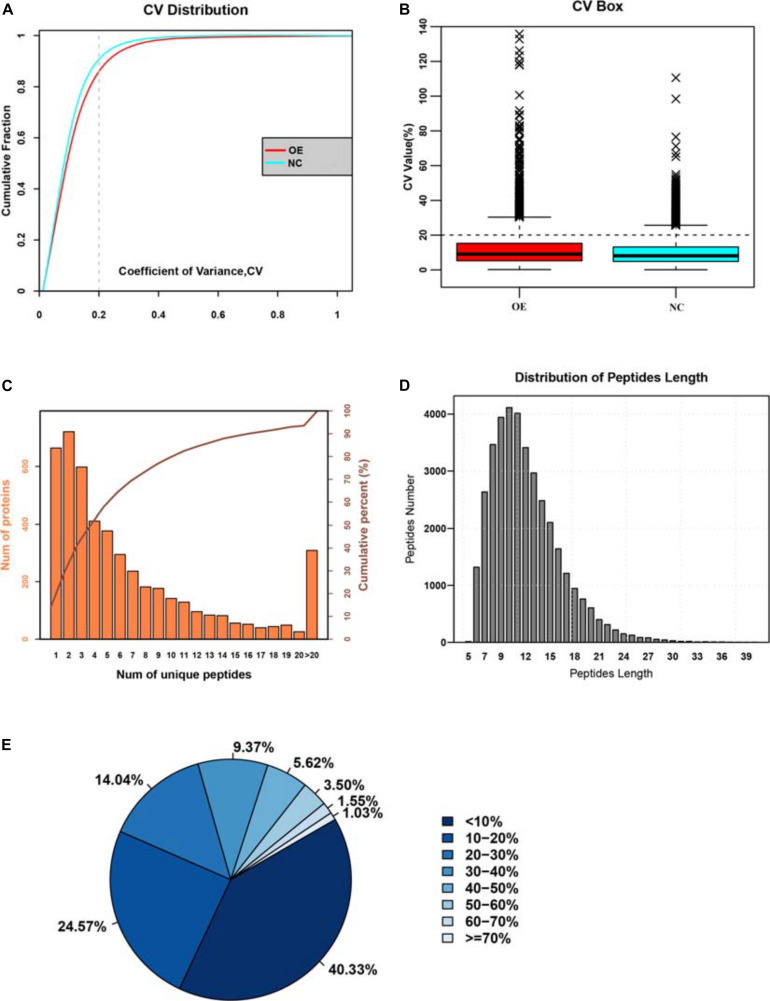
Identifying the large-changes in cellular proteome due to ACE2 overexpression. **(A)** Cumulative probability distribution of the coefficient of variation (COV) for both normal control and ACE2 overexpressing (OE) cells. **(B)** Box plot analysis of COV between NC and OE cells. **(C)** Histogram analysis showing the frequency distribution of unique peptide numbers in terms of the number of proteins they corresponded to. **(D)** Histogram analysis showing the frequency distribution of peptide length in the dataset. **(E)** Pie chart analysis showing the percentage distribution of peptide lengths.

To explore the mechanisms behind the anti-tumorigenic functions of ACE2, we decided to conduct a quantitative proteomics-based analysis for comparison between the two groups of cells in triple replicates. We used the isobaric labeling method followed by mass spectrometry (iTRAQ) to identify the changes in protein expression and signaling pathways. Affinity purification followed by mass spectrometry analysis (AP-MS) and statistical modeling of the MS1-level quantitative data allowed us to annotate the spectra, and identify the peptide species. These spectra were then matched with the known spectra to obtain a set of unique spectra, corresponding to unique proteins. The mass spectrometry data was analyzed with the ProteinPilot software using the Paragon algorith (Shilov et al., 2007) and peptides were filtered with false discovery rates (FDR value) <1% for further analysis. In total, 157,430 spectra were identified from the iTRAQ analysis and after matching with the known spectra 4,107 turned out to be unique spectra, corresponding to 4,771 proteins. A comparison of the coefficient of variation (COV, an indicator of the dispersion of data around the mean) between the two datasets and their cumulative probability distributions revealed that the two populations were significantly different, with ACE2 overexpression introducing a greater extent variation into the unique peptide dataset (Figures 3A,B). This difference in variance indicated that ACE2 overexpression alters the cellular proteome significantly by changing the expression of multiple genes, which we tried to dissect by further analysis. The distributions of unique peptides showed 4,107 proteins containing at least 2 unique peptides, accounting for 75.86% of total proteins (Figure 3C). The average length of the peptides was 15.45 and mainly concentrated between 7 and 20 (Figure 3D). The average coverage of proteins was 21.18%. The percentages of proteins with 0–10% and ≥20% of coverage were 39.12 and 38.36%, respectively, indicating higher reliability of the detected proteins in the present study (Figure 3E).

**FIGURE 4:**
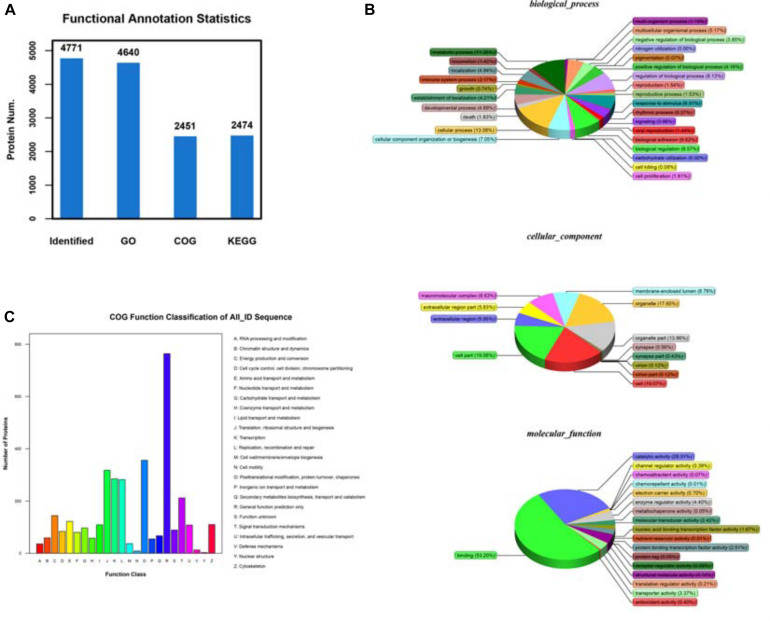
Identifying the differentially expressed proteins (DEPs) between control and ACE2 overexpressing cells. **(A)** Protein ratio distribution analysis showing the distribution of the Log_2_ fold change values of various DE peptides. **(B)** Volcano plot analysis showing the scattered distribution of Log_2_ fold change values compared to their corresponding *P*-value (–Log_10_) for each peptide. **(C)** Heat map plot of total protein intensities within the triple replicates of both control and ACE2 overexpression dataset. Heat maps were generated by hierarchical clustering analysis. The associated tree diagrams depict the clustering pattern, therefore, relationships between various samples.

Additionally, we thought that a robust biological and functional annotation of all these identified proteins would reveal the nature of the functions they performed inside the cell. To investigate, we determined the cellular localization and biological functions of the identified peptides using thorough bioinformatics-based analysis. First, to obtain insight into their biological functions, we mapped all the unique peptides with their gene ontology (GO) terms against the NCBInr animal database, using the localized BLASTP algorithm. Additionally, KEGG pathway enrichment analysis was done for gaining further insight into their functions. For the functional annotation of genes from new genomes, the system of clusters of orthologous groups of proteins (COG) was used (Figure 4A). Analysis of biological processes revealed that a large majority of all the identified unique peptides were associated with metabolic and cellular functions (Figure 4B). Further, the cellular component analysis suggested that the peptides shared a heterogeneous spatial distribution across the entire cell. While some peptides showed cell-wide distributions, others were more restricted in certain organelles, cellular, or extracellular loci (Figure 4B). Remarkably, the characterization of molecular functions indicated that a large majority of the identified peptides were either associated with binding functions or with enzymatic activity (Figure 4B). The results of the COG database mapping showed a similar functional profile for the identified unique peptides (Figure 4C). We have mapped the differentially expreesed proteins among NC and OE and categorized according to their cellular function this detailed report can be found at Supplementary Table 1: Data file. These observations, though interesting but not entirely surprising as the detected peptides performed a range of biological and molecular functions that might be critical for the survival and proliferation of A549 cells. The above pieces of evidence also establish that our pipeline protocol of mass spectrometry experiments followed by the bioinformatics analysis can reliably detect the proteins that are differentially expressed upon ACE2 overexpression and provide us with valuable insight into the mechanisms of ACE2 function.

### Identifying the Differentially Expressed Proteins (DEPs) and Annotating Their Biological Functions

**FIGURE 5:**
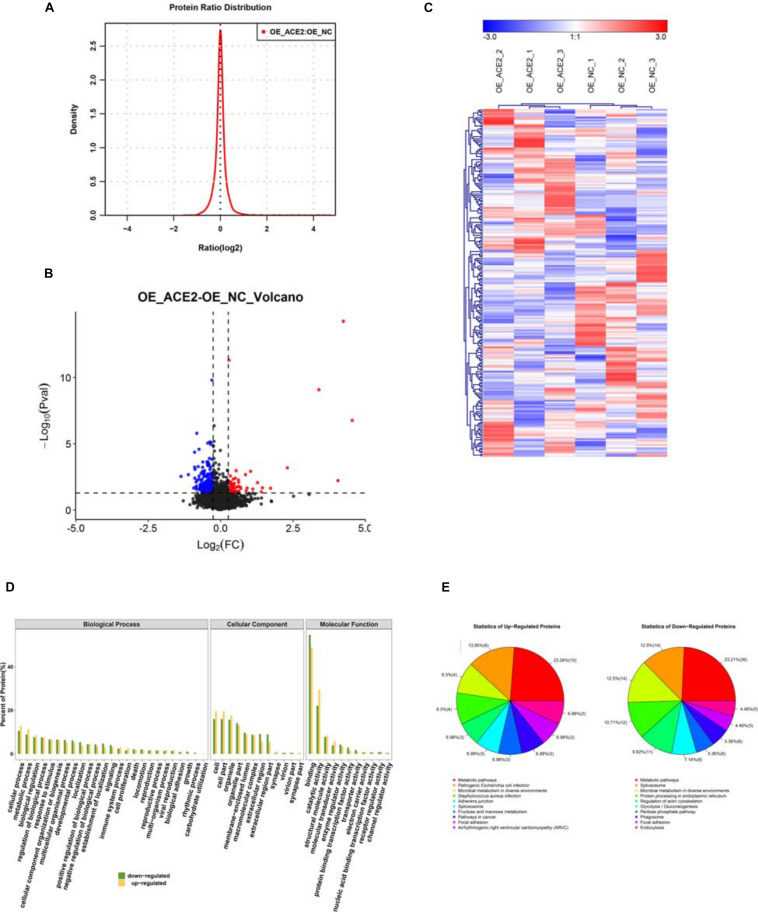
Functional annotation of the DE proteins. **(A)** Bar graph analysis showing the results of functional annotation of the DE proteins. **(B)** Pie charts showing the percentage distribution of DE proteins based on their association with various biological processes, cellular components, and molecular functions. **(C)** Histogram analysis showing the categorization of upregulated and downregulated proteins based on their association with various biological processes, cellular components, and molecular functions. **(D)** Histogram depicting the outcomes of the COG functional classifications of DEPs. **(E)** Pie charts depicting the percentage distributions of upregulated and downregulated proteins associated with various biological processes.

The high-quality data quantification from the mass spectrometry experiments allowed us to compare the datasets obtained from NC and OE cells, to discern the global proteomic changes due to ACE2 overexpression. We kept stringent criteria of fold change >1.5 fold and *p*-value < 0.05, obtained from Student’s *t*-test, to select the candidates from the screen. Out of the total of 227 proteins that were differentially regulated between vector-controlled and ACE2 overexpressing cells (ACE2/control), 70 proteins were upregulated and 147 proteins were downregulated. The protein ratio distribution plot in Figure 5A shows the distribution of (Log_2_) fold change values for all the unique peptides. The volcano plot in Figure 5B compares these (Log_2_) fold change values with their respective *P* (−Log_10_) values to obtain the distributions of differentially expressed proteins (DEPs) (both up and downregulated). Besides, from 6 samples we also generated heat map plots of “hierarchical clustering analysis” (HCA) of the total protein intensities, which helped us to appreciate the detailed alterations in the proteome (Figure 5C). As indicated by the “tree” diagram above the heatmaps, the control replicates formed a separate cluster from the ACE2 overexpression replicates suggesting a profound proteome-wide alteration.

We know from our experiment and others that the ACE2 as being one of the key regulator, upon overexpression of ACE2 we could detect large-scale changes in A549 cell proteome composition. These changes acted as positively in providing the balanced proteome and this has translated into its anti-tumorigenic functions. Consequently, we carried out bioinformatics analysis on the DEPs following the previously described pipeline protocol. Analysis of biological pathways revealed that the majority of DEP functions were associated with various cellular processes such as cell signaling, DNA replication, transcription, and post-transcriptional mechanisms (Figure 5D). Remarkably, the analysis also highlighted large-scale alterations in various metabolic pathways upon the elevation on ACE2 levels (Figure 5D). Interestingly, both the “unique peptide” dataset as well as the DEP dataset (Figures 4B, 5D) showed a similar enrichment profile for the biological pathways. Besides, the cellular component analysis of DEPs uncovered similar trends as with the entire “unique peptide” dataset. The DEPs showed a heterogeneous distribution across various cellular compartments (Figure 5D). While some of the DEPs were associated with multiple intracellular and extracellular regions with overlapping distributions, others were restricted to particular cellular loci (Figure 5D). Additionally, the molecular function analysis of DEPs established that the majority of them performed binding functions and enzymatic activity with a fraction of them carrying out other housekeeping tasks (Figure 5D). Even a separate analysis of up and downregulated genes also indicated that in both cases DEP functions were associated with various metabolic reactions (Figure 5E). However, it should be noted that multiple other pathways were affected by ACE2 overexpression as well, making it difficult to correlate ACE2 functions directly to any single biological process. Together, our extensive analysis revealed that ACE2 overexpression brought about changes in the functions of important binding molecules and enzymes associated with metabolic pathways and gene regulatory networks.

**FIGURE 6:**
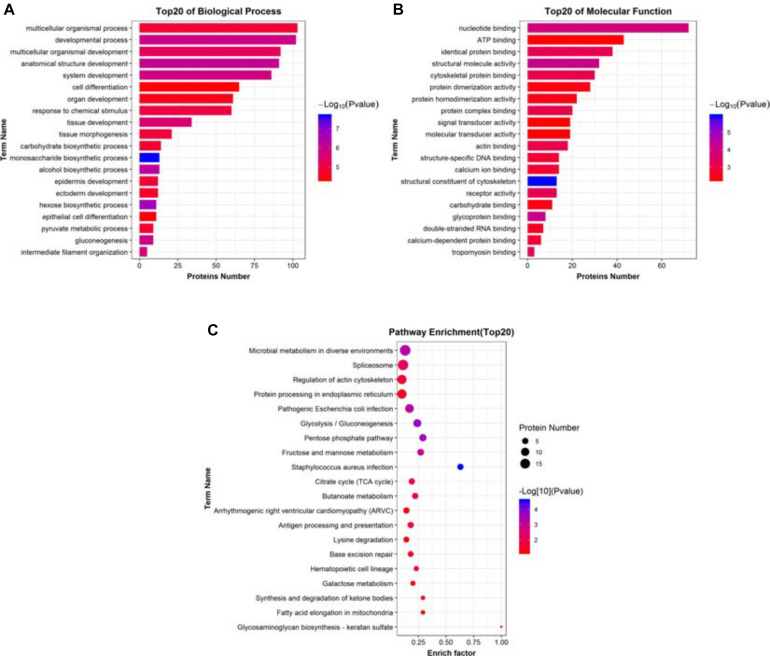
Identifying the significantly impacted pathways. **(A)** Histogram of top 20 significantly impacted biological processes DEPs associated with. **(B)** Histogram of top 20 significantly impacted molecular functions DEPs associated with. **(C)** Dot plots showing the distribution of various DEPs in multiple biological pathways obtained from pathway enrichment analysis. These pathways were assorted based on the enrich factor and the *P*-value (–Log_10_) associated with each pathway.

From the previous discussion, it was clear that overexpressing ACE2 in A549 lung cancer cells ensued a set of complex changes that ultimately led to its anti-tumorigenic potential. Therefore, to get a clearer picture of the mechanism of ACE2 function, we decided to categorize the list of the top 20 most significantly impacted biological pathways, cellular components, and molecular functions. Biological pathways were categorized based on the maximum number of proteins with various enrichment factors in combination with the *p*-values associated with each pathway. Most of the enriched pathways were associated with a variety of metabolic pathways with high confidence, corroborating our previous findings (Figure 6A). Similarly, a list of top 20 molecular component analysis showed the DEPs were associated with cytosolic non-membrane bound organelles while a strong association with various extracellular compartments were observed as well (Figure 6B). Finally, listing the top 20 candidates from molecular functions revealed a large number of proteins had nucleotide-binding ability while other functional groups included protein-protein binding, signal transduction, various Ca^2+^-dependent interactions, DNA, and RNA binding proteins (Figure 6C). Further, we have done the signaling network analysis (Supplementary Figure 2), we have found that many of the key proteins and signaling molecules like mTOR, chaperones such as HSP’s B1, A5 and A9 and RNA binding proteins have got influenced by the overexpression of ACE2.

### ACE2 Regulates the Expression of Multiple Target Genes at the Transcriptional Level

**FIGURE 7:**
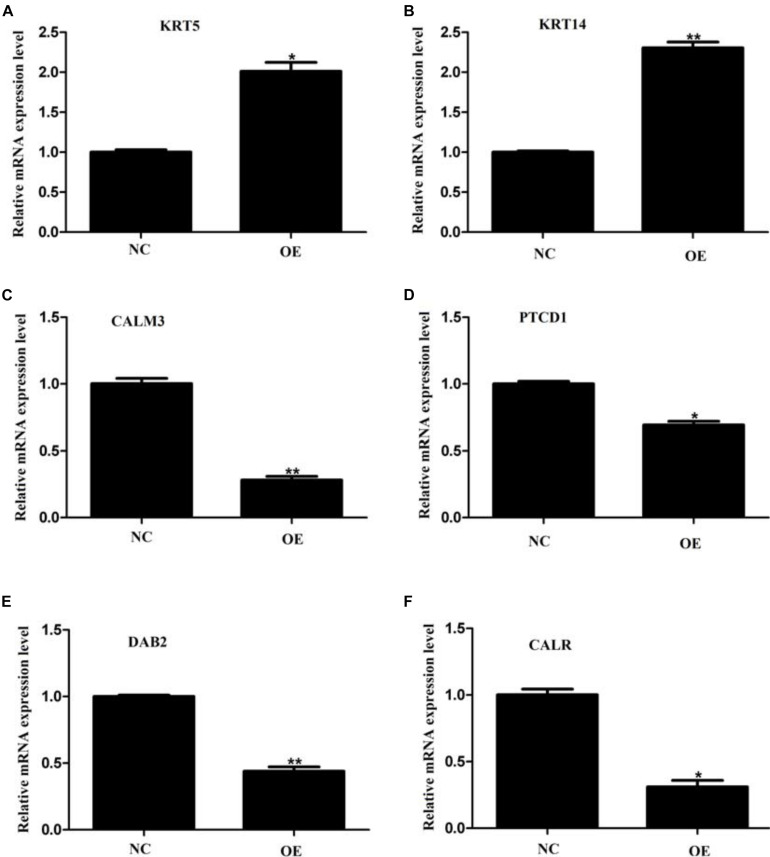
Validation of proteomic data by quantitative PCR. **(A–F)** Quantitative PCR analysis of *KRT5* **(A)**, *KRT 14* **(B)**, *CALM3* **(C)**, *PTCD1* **(D)**, *DAB2* **(E)**, and *CALR* **(F)** mRNAs on overexpression of ACE2 protein as compared to control vector. Values are normalized to those from cells with a control vector. *n* = 3, paired student’s *t*-test, ± SD. *p < 0.05, **p < 0.01.

We next decided to understand whether we could capture the changes observed in the mass spectrometry data at the mRNA level as well. To do so, we investigated the mRNA levels of six proteins that featured in the list of our DEPs. We chose two highly upregulated genes *KRT5* and *KRT 14*, which showed more than tenfold elevation in the expression level in our mass spectrometry dataset. Additionally, we selected four downregulated genes, *CALM3, PTCD1, DAB2*, and *CALR*, whose expression plummeted almost twofold in our proteomic analysis. Upon qPCR-based quantification, we observed that both *KRT5* and *KRT14* mRNAs were significantly upregulated (Figures 7A,B) while the other four mRNA levels were significantly downregulated (Figures 7C–F). This data agrees nicely with our proteomic data and highlights the reliability and robustness of our detection method. The observation also implies that ACE2 brings about the changes in the expression level of some of its target proteins by affecting their transcription. Although further investigations would be required to explore other mechanisms that might be involved in the ACE2-mediated regulation of proteome.

## Discussion

ACE2 is a relatively new member of the RAS family that serves to counter the canonical effector peptides such as Ang II (Paz Ocaranza et al., 2020). However, the enzyme has drawn extensive attention since 2003 because of the findings that it can act as the receptor for SARS Coronaviruses. Both SARS-CoV 1 and 2 hold the ability to bind to ACE2, thus making it an active node of current studies (Dai et al., 2020; Yang et al., 2020). Besides, previous studies have reported that ACE2 levels need to be actively maintained, especially in the lung, to combat inflammatory diseases (Sodhi et al., 2019). However, ACE2 is expressed in a panoply of tissues where any major perturbation of its function alters the pro and anti-inflammatory balance of the tissue, therefore, causing damage (Chai et al., 2020; Dai et al., 2020; Ng et al., 2020). For example, multiple studies have shown that upon SARS-CoV 2 entry into a cell, it severely restricts the expression of ACE2, thus causing injuries in a variety of tissues due to uncontrolled inflammation (Glowacka et al., 2010). Additionally, many studies have reported critical implications of ACE2 regulation in cancer development (Djomkam et al., 2020). For example, SARS-CoV 2 infection has been observed to increase the propensity of developing renal and intestinal cancers (Dai et al., 2020). Previous studies from A549 cells have established that ACE2 overexpression inhibits the epithelial to mesenchymal transition (EMT), a key mechanism that drives the metastatic potential of cells. The authors also show that ACE2 alters the level of TGF-β1 signaling, which, in turn, controls the expression of various epithelial and mesenchymal markers like E-cadherin, vimentin, and α-smooth muscle actin (SMA) (Qian et al., 2013). Also, ACE2 inhibits the VEGFa/VGFR2/ERK1 pathway in both lung and breast cancer cells, which reduces the angiogenic potential of the cells in both cases (Feng et al., 2010; Zhang et al., 2019). In a similar line, our current study showed that overexpressing ACE2 reduced the growth rate of the A549 cells and elevated the level of apoptosis. The above studies indicated that ACE2 overexpression reduced the migratory potential of lung cancer cells, and we also observe that ACE OE cells have the decrease in branch point and capillary length which is a indicator of anti-angiogenesis. These pieces of evidence, together, establish that ACE2 has a robust anti-tumorigenic and pro-apoptotic function.

Although these previous studies identified a few crucial signaling pathways, which were regulated by ACE2, considering the multifaceted nature of its functions, we opined that ACE2 overexpression might have induced large-scale changes in the cellular proteome. Hence, to quantify these changes, we performed the mass spectrometry-based proteomic analysis. This large-scale, unbiased, quantitative approach helped us to identify a large number of candidate proteins, whose expression levels altered due to ACE2 overexpression. A total of 227 genes showed an altered expression pattern compared to the control cells, which we dubbed as DEPs. Remarkably, our cluster analysis established that consistent proteome-wide changes occurred due to elevation in ACE2 levels. We could successfully quantify these changes by analyzing the coefficient of variation between the two datasets. The data further helped us to predict the significantly impacted biological processes, cellular component, and molecular function analysis. Matching with gene ontology terms, functional analysis, and pathway enrichment analysis revealed that DEPs were mostly associated with multiple metabolic and gene regulatory cellular processes. While the gene regulatory processes are predicted targets of a protein that regulates cell growth and proliferation, it was rather interesting to observe a global change in the protein levels related to metabolism. For example, pathway enrichment analysis indicated that a reduction in glycolysis and an increase in the pentose phosphate pathway (PPP) accompanied ACE2 overexpression. Glycolysis and PPP are two major pathways by which a glucose molecule is metabolized to produce energy or other metabolic intermediates. It has been observed before that various cancer cells prefer to utilize glucose anaerobically through glycolysis due to the “Warburg effect” that allows them to have easy availability of ATP (DeBerardinis and Chandel, 2020). Intriguingly, our analysis showed that an increased ACE2 level can counter this effect by reducing the protein levels related to glycolysis. Other relevant pathways that were picked up in our bioinformatics analysis included lipid, fatty acid, amino acid, and other forms of carbon metabolic pathways. These results, therefore, underscores the importance of orchestrating metabolic networks as a critical feature of ACE2 functionality. Further experiments and analysis at the level of metabolomics would help to uncover the specific changes induced by ACE2 overexpression.

Also, the DEPs showed a heterogeneous cell-wide distribution in the cellular component analysis. This isn’t surprising considering eclectic ACE2 functions and significant cellular changes it can introduce, such as preventing EMT or reducing the extent of their migration. Additionally, molecular function analysis revealed a large number of the DEPs had a nucleotide-binding function, DNA binding, and RNA binding ability. Combining these results with the previously described pathway analysis suggest that these DEPs were mostly associated with spliceosome functions and DNA damage repair system. Besides, the second-largest represented group of proteins were found to have enzymatic activities that might be linked to various signaling pathways, metabolic conversions, and cellular housekeeping functions such as remodeling actin cytoskeleton. Finally, from the list of DEP candidates, we verified the mRNA expression levels of 6 of them, 2 of which were upregulated and 4 that were downregulated on ACE2 overexpression. These results also validated that a substantial part of ACE2-mediated changes involved transcriptional regulation of genes.

In summary, we could show that ACE2 overexpression reduced the rate of cell proliferation and increased the extent of apoptosis in A549 lung cancer cells through large-scale alterations in protein levels that are associated with metabolic pathways and gene expression regulatory networks.

## Materials and Methods

### Cell Culture

The lung cancer cell line (A549) was obtained from the Shanghai Cell Bank of the Chinese Academy of Sciences (Shanghai, China). Cells were cultured in Dulbecco’s modified Eagle medium (Gibco, United States) supplemented with 10% fetal bovine serum (Gibco, United States), 100 U/ml penicillin, and 100 μg/ml streptomycin, and incubated at 37°C under 5% CO_2_.

### Plasmid Construction, Lentivirus Package and Infection of A549 Cells

*ACE2* CDS was cloned in the pcDNA3.1 (+) vector using the restriction sites for *Kpn*I (GGTACC)-*Xho*I (CTCGAG) with an insert size of 2,337 bp. This insert was validated by sequencing. Two microgram of ACE2 or empty plasmid was mixed with other lentivirus packaging plasmids pMDLg-pRRE, pMD2.G, and pRSV-Rev following the standard protocol (Cribbs et al., 2013). To verify the titer of the lentivirus, quantitative polymerase chain reaction (qPCR) detecting Gag-specific primers was performed. A549 cells were further infected with 20 multiplicity of infection (MOI) lentivirus for 24 h and incubated in fresh medium.

### RNA Extraction and Real-Time PCR

Total RNA was extracted by using TRIzol (Ambion). The RNA was further purified with two phenol-chloroform treatments and then treated with RQ1 DNase (Promega) to remove DNA. The quality and quantity of the purified RNA were determined by measuring the absorbance at 260 nm/280 nm (A260/A280) using Smartspec Plus (BioRad). The integrity of RNA was further verified by 1.2% agarose gel electrophoresis. Real-time quantitative PCR (RT-qPCR) was performed for detecting gene expressions using the primers described below (Table 1). cDNA synthesis was done by standard procedures and real-time PCR was performed on the Bio-Rad S1000 (BioRad) with Bestar SYBR Green RT-PCR Master Mix (DBI Bioscience, Shanghai, China). The PCR conditions consist of denaturing at 95°C for 10 min, 40 cycles of denaturing at 95°C for 15 s, annealing, and extension at 60°C for 1 min. qPCR amplifications were performed in triplicates for each sample. Transcript levels for the genes analyzed were measured in comparison with the housekeeping gene *actin* as an internal reference standard, using the 2^–ΔΔCT^ method (14) (Livak and Schmittgen, 2001).

**TABLE 1:** The genes and primers used for qRT-PCR experiments.

Gene	Forward primer (5′–3′)	Reverse primer (5′–3′)
*KRT5*	TGATGCTGAAGAAGGATGTAGATGC	CCAGGTTGCGGTTGTTGTCC
*KRT14*	CCTGGCGTGGACCTGAGC	GAAGAACCATTCCTCGGCATCC
*CALM3*	GGGAACGGGACCATTGACTTC	TCATCTCATCCACCTCCTCATCG
*PTCD1*	AGACGAGGAGGAGGAGGAGAG	GGAACTGGGCTGCGGATTTG
*DAB2*	TTTGAGTGCCTTTGCCAGTTATTTC	CAGGTTGAGAAGAAGCCACAGAG
*SOD2*	ACAAAGGCAGCAGAGAAACAAATG	GTCCTCCTCATCCTCCTCATCC
*Actin*	TGGACTTCGAGCAAGAGATG	GAAGGAAGGCTGGAAGAGTG

### Western Blotting Analysis

Cultured lung cancer cells were harvested and homogenized with cell lysis buffer. Then, the homogenates were centrifuged for 30 min at 4°C, 12,000 rpm, and the supernatants were collected as protein samples. Protein amounts were measured using the BCA Protein Assay Kit (Beyotime, China). Equal amounts of protein samples were separated by denaturing 10% SDS-PAGE and transferred onto polyvinylidene difluoride (PVDF) membranes. Membranes were incubated in a 5% skim milk TBST blocking solution at room temperature (RT) for 1 h and membranes were incubated with agitation at 4°C overnight with specific primary antibodies anti-ACE2 and anti-GAPDH (Beyotime, China), anti-Bax (Cell signaling technology, cat.no-2772S, anti-P53 (Proteintech, cat.no-10442-1-AP). Then, membranes were incubated by secondary antibodies conjugated with horseradish peroxidase (HRP) (Zhong san jinqiao, Beijing, China) at RT for 50 min. Finally, protein bands were visualized using an enhanced chemiluminescence (ECL) Western blotting detection system (GE Healthcare, Amersham, United Kingdom).

### Cell Viability Detection

The proliferation of lung cancer cells was evaluated using cell counting Kit-8 (Beyotime, China). A549 cells were transferred into a 96-well cell culture plate, with 200 μl suspension per well, and grown overnight. At 24, 48, and 72 h, 20 μl CCK-8 was added to each well, and then the plates were incubated for 2 h. Finally, absorbance was measured at 490 nm with a microplate reader (Bio-Rad). All groups were performed in quintuplicate.

### Flow Cytometric Detection

Apoptosis in lung cancer cells was determined by flow cytometry using Annexin V-conjugated FITC Apoptosis detection kit (BD, Franklin Lakes, NJ, United States). Briefly, after infection lentivirus for 48 h, cells were harvested, washed twice with PBS, and incubated with Annexin V-FITC and PI for 10 min in the dark. Then, the stained cells were detected using MoFLO XDP (Beckman).

### Transwell Invasion

For the invasion assay, chambers were assembled in 24-well plates 8 μm pore transwell inserts (BD Falcon, Franklin Lakes, NJ, United States). Inserts were coated with 50 μl Matrigel (diluted 1:4 in serum-free media). Lung cancer cells (A549) (10^5^) were placed in the upper chamber. Invaded cells on the underside of the inserts were fixed with 4% paraformaldehyde and stained with 0.1% crystal violet. Images were captured using a stereomicroscope (Leica, Wetzlar, Germany).

### Tube Formation Assay

For this assay we grown A549 cells in logarithmic growth phase and transfected with pcDNA3.1 empty and pcDNA3.1-ACE2 plasmid for 48 h. After centrifugation (2,000 rpm/min, 20 min) supernatant was collected after 2–3 centrifugations, collected fractions were aliquoted to 1.5 ml tube for later usage and stored at −20°C. 96-well plate, spare cell supernatant, 200 ul pipette tip, and pipette were kept in refrigerator at 4°C for 20–30 min. Further, Matrigel was thawed at 4°C. Fifty microliter/well of Matrigel was added to 96-well plate, care must be taken not to leave any air bubbles. Plate was kept in the cell culture incubator for solidification for 30 min. One milliliter trypsin containing EDTA was added to digest HUVEC cells, later serum-containing culture medium was added to stop the digestion. Cell count was done and cell density of 1^∗^104 cells/ml was adjusted. Once the gel has solidified, 50 ul of cell suspension was added to 96-well plate and cells were incubated for 4–6 h and cell images were taken under microscope. Cells were analyzed and quantified for vascular endothelial cell tubule branches and tube length.

### iTRAQ Quantitative Proteomics Analysis

The cells (5 × 10^6)^ were lysed in lysis buffer (7M urea, 2M thiourea, 4% sodium dodecyl sulfate, 40 mM Tris-HCl, pH 8.5) containing 1 mM phenylmethanesulfonyl fluoride (PMSF) and 2 mM ethylenediaminetetraacetic acid (final concentration) for 5 min. The lysate was then sonicated for 10 min on ice and then centrifuged at 4°C, 13,000 g for 20 min following which, the supernatant was mixed with four volumes of precooled acetone at −20°C overnight. After centrifugation, the protein pellets were air-dried and resuspended in 8M urea/100 mM triethylamine borane (TEAB) (pH 8.0). Protein samples were then reduced with 10 mM DL-Dithiothreitol (DTT) at 56°C for 30 min and alkylated with 50 mM iodoacetamide (IAM) at room temperature for 30 min in the dark. After diluting with 10 mM TEAB four times, the total protein concentration was measured using the Bradford method (Bradford, 1976). Equal amounts of proteins from each sample were used for tryptic digestion. Trypsin was added at an enzyme-protein ratio of 1:50 (wt/wt) and the digestion reaction was performed at 37°C for 12–16 h. Following the digestion protocol, peptides were desalted using C18 columns and they were dried with a vacuum concentration meter. The dried peptide power was further redissolved with 0.5M TEAB to obtain a volume of 20 μl for peptide labeling. Samples were labeled with the iTRAQ Reagent 8PLEX Multiplex Kit (AB Sciex U.K. Limited, Shanghai, China) according to the manufacturer’s instructions. Samples were iTRAQ labeled as follows: NC-1, X1; NC-2, X2; NC-3, X3; ACE2-1, X4; ACE2-2, X5; and ACE2-3, X6. All of the labeled samples were mixed with equal amounts. Next, the labeled samples were fractionated using a high-performance liquid chromatography system (Thermo Dionex Ultimate 3000 BioRS, Thermo Fisher Scientific, Waltham, MA) using a Durashell C18 (Bonna-Agela Technologies Inc., Wilmington, DE) (5 μm,100 Å,4.6 × 250 mm) under high-pH conditions. Finally, the collected fractions were combined into 12 fractions. The peptide samples were dissolved in 2% acetonitrile-0.1% formic acid and analyzed using a TripleTOF 5,600 + mass spectrometer coupled with the Eksigent NanoLC System (SCIEX). Peptides were loaded onto a C18 trap column (5 μm, 100 μm × 20 mm) and eluted at 300 ml/min onto a C18 analytical column (3 μm, 75 μm × 150 mm) over a 90 min gradient. The two mobile phases consisting of buffer A (2% Acetonitrile-0.1% formic acid–98% H_2_O) and buffer B (98% acetonitrile-0.1% formic acid-2% H_2_O) could be observed. For information-dependent acquisition (IDA), survey scans were acquired in 250 ms, and 30 production scans were collected in 100 ms per scan. MS1 spectra were collected in the range 350–1,500 m/z and MS2 spectra were collected in the range 100–1,500 m/z. Precursor ions were excluded from reselection for 15 s. In the IDA advanced tab, the option “Adjust CE when using iTRAQ reagent” was selected for iTRAQ samples.

The original MS/MS file data were analyzed using ProteinPilot Software v4.5 (AB Sciex, Shanghai, China). For protein identification, the Paragon algorithm (Shilov et al., 2007), which was integrated into ProteinPilot, was used against the UniProt/SwissProt database for database searching. The parameters were set as follows: The instrument was TripleTOF 5,600 + iTRAQ quantification, and cysteine modified with IAM; biological modifications were selected as ID focus, trypsin digestion, the quantitate, bias correction, and background correction was used for protein quantification and normalization. For calculation of the false discovery rate (FDR), an automatic decoy database search strategy (Choi et al., 2008) was used to estimate FDR using the proteomics system performance evaluation pipeline software (PSPEP, integrated into the ProteinPilot Software). Unique peptides were used for iTRAQ labeling quantification, and peptides with global FDR values from fit less than 1% were considered for further analysis. Within each iTRAQ run, DEPs were determined based on the ratios of differently labeled proteins and *p*-values provided by ProteinPilot; the *p*-values were generated by ProteinPilot using the peptides used to quantify the respective protein. For the determination, of DEPs, fold changes were calculated as the average comparison pairs among biological replicates. Proteins with a fold change larger than 1.2 and a *p* < 0.05 were considered to be changes that are significantly different.

### Bioinformatics and Annotations

To determine the biological and functional properties of all the identified proteins, the identified protein sequences were mapped with gene ontology (GO) terms^1^. For this, a homology search was first performed for all the identified sequences with a localized NCBI BLASTP program against the NCBInr animal database. The “e” value was set to less than 1e-5, and the best hit for each query sequence was taken into account for GO term matching. The GO term matching was performed using the blast2go v4.5 pipeline. The system of clusters of orthologous groups of proteins (COG^2^) was used for the functional annotation of genes from new genomes and research into genome evolution.

### Statistical Analyses

The data were analyzed for statistical significance using Microsoft Excel (2010). All data are presented as the mean ± standard deviation (mean ± SD). Student’s *t-*test (paired) was used to check the statistical significance while comparing the means of two data sets. For the cases with a *p*-value < 0.05 was considered as a statistically significant difference.

## Data Availability Statement

The datasets presented in this study can be found in online repositories. The names of the repository/repositories and accession number(s) can be found below: ProtemXchange, Accession No: PXD024899.

## Author Contributions

KX, FX, and LX conceived of the study and participated in its design. LS and YB participated in the design of the study and coordination and drafted the manuscript. PL and YL participated in the design of the study assessed treatment effectiveness and collected clinical data. KX and LS participated in the design of the study, performed the statistical analysis, and drafted the manuscript. All authors read and approved the final manuscript.

## Conflict of Interest

The authors declare that the research was conducted in the absence of any commercial or financial relationships that could be construed as a potential conflict of interest.

## Footnotes

1 http://geneontology.org/

2 http://www.ncbi.nlm.nih.gov/COG/

## Supplementary Material

Supplementary Figure 1 Establishment of ACE2 overexpression cell line and characterization of its growth rate. **(A)** Immunoblotting for Bax and P53 protein from control and ACE2 overexpressing cells with GAPDH as a loading control. **(B,C)** quantification of replicate data for P53 and Bax protein (*N* = 3 and ± SD, *T*-test *p*-value < 0.01).

Supplementary Figure 2 Identifying the significantly impacted pathways. Bioinformatics analysis of the mass spectrometry data indicating the signaling network analysis.

Supplementary Table 1 Data file: Differentially expressed protein across the NC and ACE2 OE cells.

## References

[B1] Bradford M. M. (1976). A rapid and sensitive method for the quantitation of microgram quantities of protein utilizing the principle of protein-dye binding. *Anal. Biochem.* 72 248–254. 10.1016/0003-2697(76)90527-3 942051

[B2] Cai L. Qin X. Xu Z. Song Y. Jiang H. Wu Y. (2019). Comparison of cytotoxicity evaluation of anticancer drugs between real-time cell analysis and CCK-8 method. *ACS Omega* 4 12036–12042. 10.1021/acsomega.9b01142 31460316 PMC6682106

[B3] Chai P. Yu J. Ge S. Jia R. Fan X. (2020). Genetic alteration, RNA expression, and DNA methylation profiling of coronavirus disease 2019 (COVID-19) receptor ACE2 in malignancies: a pan-cancer analysis. *J. Hematol. Oncol.* 13:43. 10.1186/s13045-020-00883-5 32366279 PMC7197362

[B4] Choi H. Ghosh D. Nesvizhskii A. I. (2008). Statistical validation of peptide identifications in large-scale proteomics using the target-decoy database search strategy and flexible mixture modeling. *J. Proteome Res.* 7 286–292. 10.1021/pr7006818 18078310

[B5] Cribbs A. P. Kennedy A. Gregory B. Brennan F. M. (2013). Simplified production and concentration of lentiviral vectors to achieve high transduction in primary human T cells. *BMC Biotechnol.* 13:98. 10.1186/1472-6750-13-98 24215295 PMC3830501

[B6] Dai Y. J. Hu F. Li H. Huang H. Y. Wang D. W. Liang Y. (2020). A profiling analysis on the receptor ACE2 expression reveals the potential risk of different type of cancers vulnerable to SARS-CoV-2 infection. *Ann. Transl. Med.* 8:481. 10.21037/atm.2020.03.61 32395525 PMC7210193

[B7] DeBerardinis R. J. Chandel N. S. (2020). We need to talk about the warburg effect. *Nat. Metab.* 2 127–129. 10.1038/s42255-020-0172-2 32694689

[B8] Djomkam A. L. Z. Olwal C. O. Sala T. B. Paemka L. (2020). Commentary: SARS-CoV-2 cell entry depends on ACE2 and TMPRSS2 and is blocked by a clinically proven protease inhibitor. *Front. Oncol.* 10:1448. 10.3389/fonc.2020.01448 32974166 PMC7466403

[B9] Feng Y. Wan H. Liu J. Zhang R. Ma Q. Han B. (2010). The angiotensin-converting enzyme 2 in tumor growth and tumor-associated angiogenesis in non-small cell lung cancer. *Oncol. Rep.* 23 941–948. 10.3892/or_00000718 20204277

[B10] Glowacka I. Bertram S. Herzog P. Pfefferle S. Steffen I. Muench M. O. (2010). Differential downregulation of ACE2 by the spike proteins of severe acute respiratory syndrome coronavirus and human coronavirus NL63. *J. Virol.* 84 1198–1205. 10.1128/jvi.01248-09 19864379 PMC2798380

[B11] Hamming I. Cooper M. E. Haagmans B. L. Hooper N. M. Korstanje R. Osterhaus A. D. (2007). The emerging role of ACE2 in physiology and disease. *J. Pathol.* 212 1–11. 10.1002/path.2162 17464936 PMC7167724

[B12] Livak K. J. Schmittgen T. D. (2001). Analysis of relative gene expression data using real-time quantitative PCR and the 2(-Delta Delta C(T)) Method. *Methods* 25 402–408. 10.1006/meth.2001.1262 11846609

[B13] Ma D. Chen C. B. Jhanji V. Xu C. Yuan X. L. Liang J. J. (2020). Expression of SARS-CoV-2 receptor ACE2 and TMPRSS2 in human primary conjunctival and pterygium cell lines and in mouse cornea. *Eye* 34 1212–1219. 10.1038/s41433-020-0939-4 32382146 PMC7205026

[B14] Ng K. W. Attig J. Bolland W. Young G. R. Major J. Wrobel A. G. (2020). Tissue-specific and interferon-inducible expression of nonfunctional ACE2 through endogenous retroelement co-option. *Nat. Genet.* 52 1294–1302. 10.1038/s41588-020-00732-8 33077915 PMC7610354

[B15] Paz Ocaranza M. Riquelme J. A. Garcia L. Jalil J. E. Chiong M. Santos R. A. S. (2020). Counter-regulatory renin-angiotensin system in cardiovascular disease. *Nat. Rev. Cardiol.* 17 116–129. 10.1038/s41569-019-0244-8 31427727 PMC7097090

[B16] Prabakaran P. Xiao X. Dimitrov D. S. (2004). A model of the ACE2 structure and function as a SARS-CoV receptor. *Biochem. Biophys. Res. Commun.* 314 235–241. 10.1016/j.bbrc.2003.12.081 14715271 PMC7117316

[B17] Qian Y. R. Guo Y. Wan H. Y. Fan L. Feng Y. Ni L. (2013). Angiotensin-converting enzyme 2 attenuates the metastasis of non-small cell lung cancer through inhibition of epithelial-mesenchymal transition. *Oncol. Rep.* 29 2408–2414. 10.3892/or.2013.2370 23545945

[B18] Shilov I. V. Seymour S. L. Patel A. A. Loboda A. Tang W. H. Keating S. P. (2007). The paragon algorithm, a next generation search engine that uses sequence temperature values and feature probabilities to identify peptides from tandem mass spectra. *Mol. Cell. Proteomics* 6 1638–1655. 10.1074/mcp.t600050-mcp200 17533153

[B19] Sodhi C. P. Nguyen J. Yamaguchi Y. Werts A. D. Lu P. Ladd M. R. (2019). A dynamic variation of pulmonary ACE2 is required to modulate neutrophilic inflammation in response to *Pseudomonas aeruginosa* lung infection in mice. *J. Immunol.* 203 3000–3012. 10.4049/jimmunol.1900579 31645418 PMC7458157

[B20] Tikellis C. Thomas M. C. Burns W. C. (2012). Angiotensin-converting enzyme 2 (ACE2) is a key modulator of the renin angiotensin system in health and disease. *Int. J. Pept.* 2012:256294. 10.1155/2012/256294 22536270 PMC3321295

[B21] Yamaguchi M. Hirai S. Sumi T. Tanaka Y. Tada M. Nishii Y. (2017). Angiotensin-converting enzyme 2 is a potential therapeutic target for EGFR-mutant lung adenocarcinoma. *Biochem. Biophys. Res. Commun.* 487 613–618. 10.1016/j.bbrc.2017.04.102 28433633 PMC7092918

[B22] Yang J. Petitjean S. J. L. Koehler M. Zhang Q. Dumitru A. C. Chen W. (2020). Molecular interaction and inhibition of SARS-CoV-2 binding to the ACE2 receptor. *Nat. Commun.* 11:4541. 10.1038/s41467-020-18319-6 32917884 PMC7486399

[B23] Zhang Q. Lu S. Li T. Yu L. Zhang Y. Zeng H. (2019). ACE2 inhibits breast cancer angiogenesis via suppressing the VEGFa/VEGFR2/ERK pathway. *J. Exp. Clin. Cancer Res.* 38:173. 10.1186/s13046-019-1156-5 31023337 PMC6482513

