# Supplementary figures and images for: Decipering the Molecular Mechanism of ACE2 Regulating A549 Cells
Source: Front Genet. 2021 Jul 19;12:653725. doi: 10.3389/fgene.2021.653725 (PMC8329961; doi:10.3389/fgene.2021.653725)

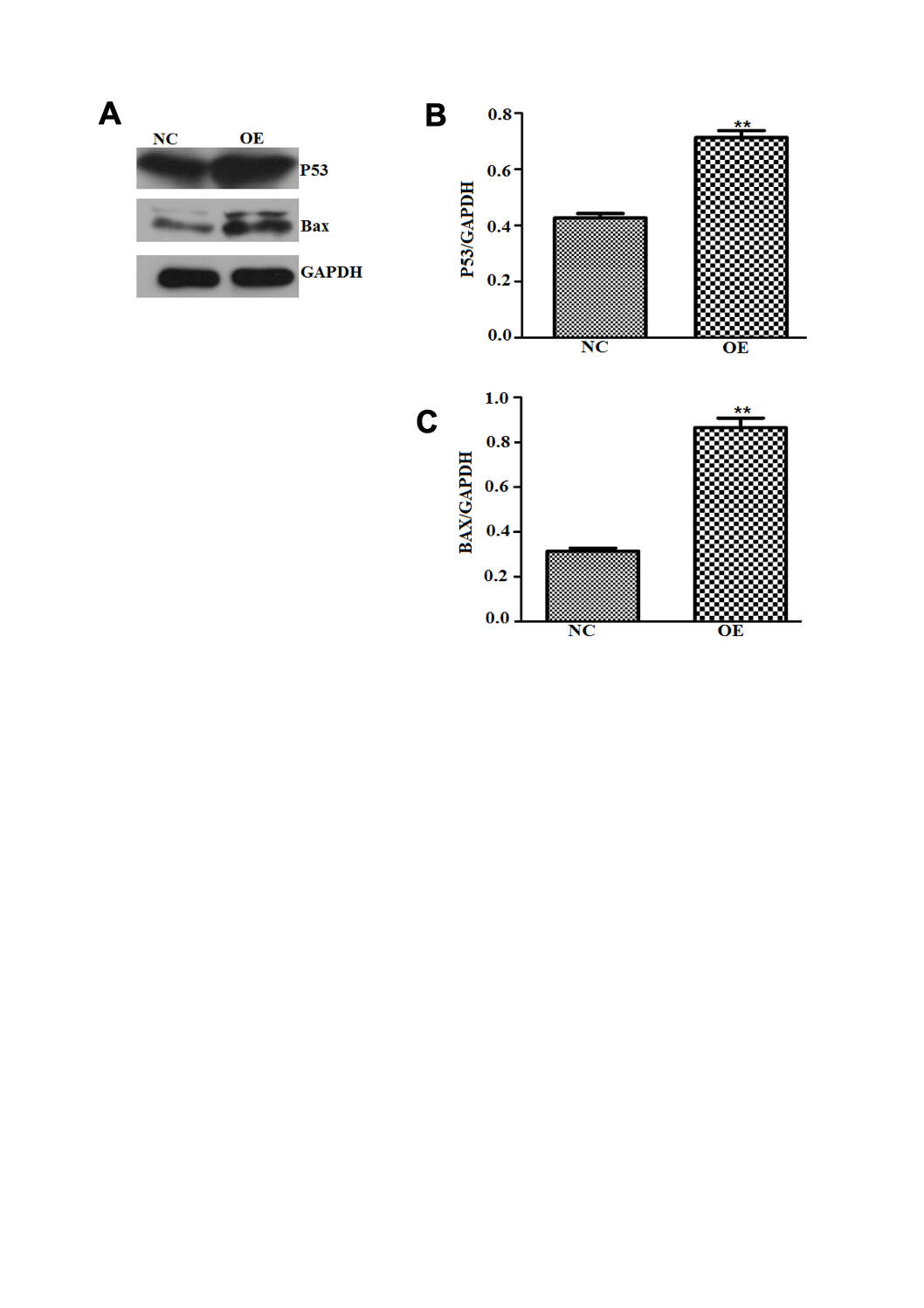

Supplement: Supplementary Figure 1 — Establishment of ACE2 overexpression cell line and characterization of its growth rate. (A) Immunoblotting for Bax and P53 protein from control and ACE2 overexpressing cells with GAPDH as a loading control. (B,C) quantification of replicate data for P53 and Bax protein (N = 3 and ± SD, T-test p-value < 0.01). [file Image_1.TIF]

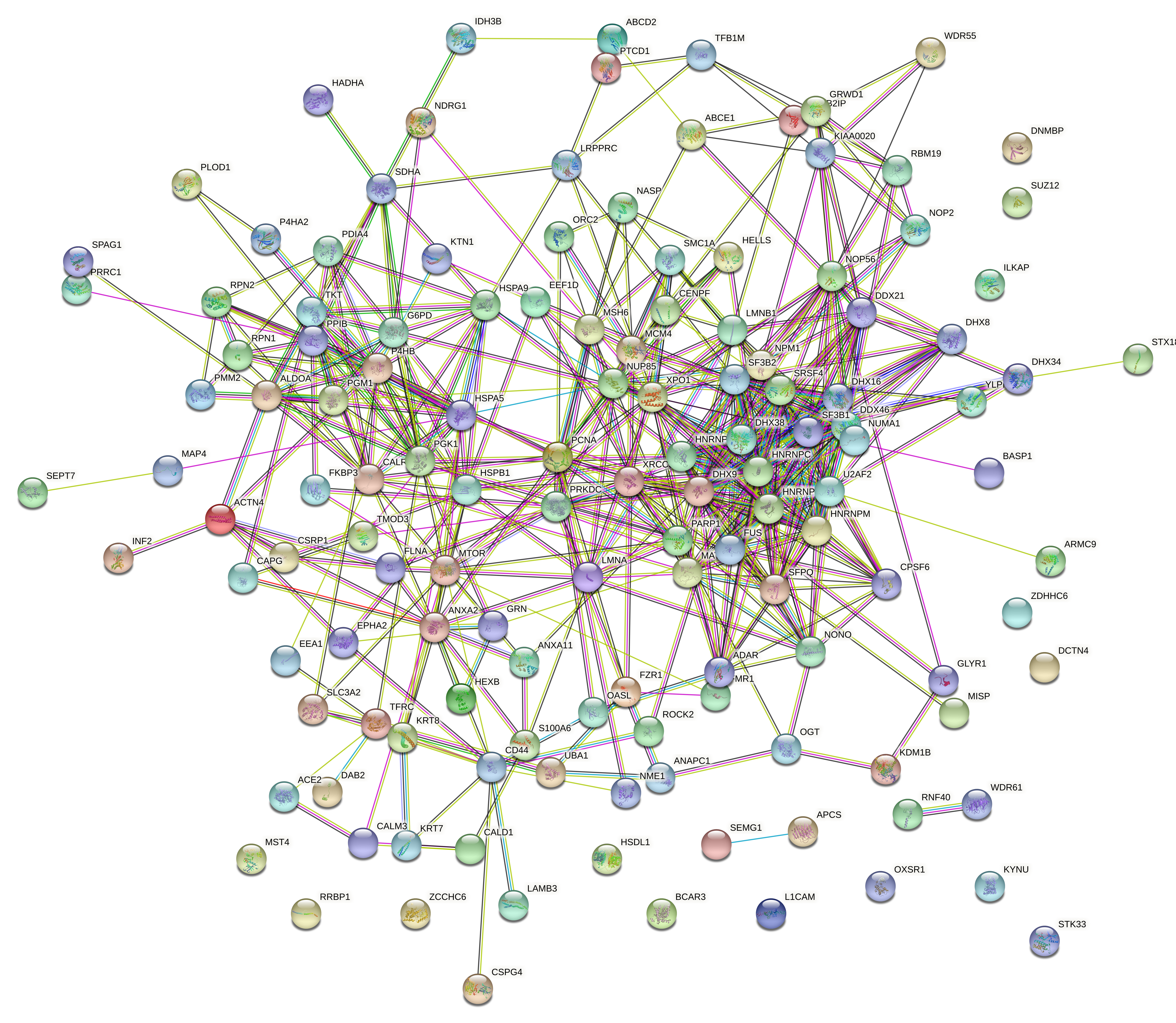

Supplement: Supplementary Figure 2 — Identifying the significantly impacted pathways. Bioinformatics analysis of the mass spectrometry data indicating the signaling network analysis. [file Image_2.PNG]
